# The Medico-Sociological Section at Brighton

**Published:** 1913-08-09

**Authors:** Reginald John Ryle

**Affiliations:** President of the Medico-Sociological Section. 15 German Place, Brighton


### THE EDITOR'S LETTER-BOX.

### PROCEDURE AND PAPERS AT BRITISH MEDICAL ASSOCIATION MEETINGS.

### The Medico-Sociological Section at Brighton.

Sir,—My attention has been drawn to the leading article in the issue of The Hospital for August 2, which is headed, "Our Hospitals: Their Future Organisation and Work." As certain criticisms are made upon the conduct of the Medico-Sociological Section at the recent annual meeting of the British Medical Association, I shall be obliged if you will give me the opportunity to reply to two of them.

It is objected that the executive failed to place the papers in the hands of those invited to take part in the discussions a few days before the meetings were held. As a matter of fact, four weeks before the meeting a syllabus of each of the papers was published, and the four and a half closely printed pages were issued to every one who had received an invitation to attend the Section. With this assistance everyone who had read the syllabus was in a position to know beforehand the line which would be taken by each reader of a paper.

The second criticism is to the effect that it had been notified that an afternoon session of the Section would be held on Friday, the 25th, but that the announcement was made at half-past twelve that there would not be an afternoon session, and much disappointment was caused in consequence.

In reply to this criticism, I may say that I was informed in the morning that, in part owing to the requirements of attendance at the Standing Committee for Amendment of the National Insurance Act, and in part to other unavoidable causes, three or four speakers who should have taken part in the discussion were prevented from coming. Accordingly the discussion was left to a smaller number, and, when the papers had been read, the question was put to those who were present whether under these circumstances they would prefer to continue the discussion with a view to completing it soon after one o'clock, or whether they would wish to postpone it to an afternoon meeting. The vote was *nem. con.* against an afternoon session. It is, of course, a matter of regret that our list of speakers was thus depleted, but the meeting decided for itself in favour of going on, against the afternoon session, and probably we should not have secured more than a very small attendance had the decision been other than it was. It is impossible by any arrangements to please all, but I can assure your readers that during the past six months my most energetic local Hon. Secretary, Dr. Rowland Fothergill, has left no stone unturned to ensure that our meetings should be worthy of the occasion, and I believe, Sir, that your generous declaration that "the Medico-Sociological Section at the meeting of the British Medical Association at Brighton was probably the most successful of all" expresses the opinion of a good many who attended the eighty-first annual meeting.—I am, yours, etc.,

Reginald John Ryle, M.D., J.P.,
President of the Medico-Sociological Section.
15 German Place, Brighton, August 4, 1913.

[Dr. Ryle's Section was full of interest throughout, but its practical value and influence might have been twice as great if the papers had been supplied as we suggested. A syllabus may prove a pitfall, and no practised speaker will base discussion upon it. So Professor Moore's able paper did not receive the notice its merits and importance demanded. His many points could not be examined or discussed without the production of the paper. Dr. Ryle was courtesy itself, but it was unfortunate that the order of business announced in the morning should have been cancelled at the eleventh hour. —Ed. The Hospital.]

